# Changes in anterior chamber volume after implantation of posterior chamber phakic intraocular lens in high myopia

**DOI:** 10.1186/s12886-018-0830-2

**Published:** 2018-07-28

**Authors:** Yi Zhu, Haobin Zhu, Yan Jia, Jibo Zhou

**Affiliations:** 1 Department of Ophthalmology, Shanghai Ninth People’s Hospital, Shanghai Jiao Tong University School of Medicine, 639 Zhizaoju Road, Shanghai, 200011 China; 2 Shanghai Key Laboratory of Orbital Diseases and Ocular Oncology, Shanghai, China; 3 Department of Ophthalmology, Children’s Hospital of Fudan University, Shanghai, China

**Keywords:** Anterior chamber volume, Implantable collamer lens, High myopia

## Abstract

**Background:**

The present study aimed to assess changes in, and the factors that influence, anterior chamber volume (ACV) after implantable contact lens (ICL) implantation in high myopia eyes using a Pentacam.

**Methods:**

The study sampled 26 high myopia patients (45 eyes) who were treated with ICL implantation. These patients were followed for an average of 4.28 months postoperatively. ACV was measured with a Pentacam preoperatively and at 3 months postoperatively. The data were analyzed by paired samples Wilcoxon signed-rank test. Generalized estimating equation (GEE) model adjusting within-patient intereye correlations in addition to Pearson’s and Spearman’s correlation tests were performed to determine associations.

**Results:**

The mean ACV was 198.33 ± 33.08 mm^3^ before surgery and 118.65 ± 17.70 mm^3^ after surgery. A significant decrease of 79.68 mm^3^ (40.18%) (*Z* = 5.841, *P* <  0.001) was detected. Positive correlations were found between ACV changes and ICL central vault (*r* = 0.528, *P* <  0.001) and preoperative anterior chamber depth (ACD) (*r* = 0.665, *P* <  0.001). There were positive correlations between postoperative ACV and postoperative anterior chamber angle (ACA) at 3:00 o’clock (*r* = 0.448, *P* = 0.002) and at 9:00 o’clock (*r* = 0.405, *P* = 0.006). GEE regression model showed that postoperative ACV significantly positively correlated with preoperative ACV (*P* = 0.002), ACD (*P* = 0.002) and horizontal ACA (*P* = 0.005) and negatively correlated with ICL central vault (*P* <  0.001).

**Conclusion:**

Complementary to vault and ACD, ACV is a sensitive parameter with certain value of preoperative assessment and postoperative monitoring in ICL implantation.

## Background

The implantable collamer lens (ICL V4; STAAR Surgical, Nidau, Switzerland) is a sulcus-placed posterior chamber phakic intraocular lens that can correct high myopia. Compared with keratorefractive surgeries, ICL V4 implantation has several advantages, including faster visual recovery, more stable refraction, better visual quality, reversibility of the surgical procedure and exchangeability of the ICL. However, ICL V4 implantation creates an artificial situation of a shallow anterior chamber and pupillary block. Therefore, laser peripheral iridectomy (LPI) is routinely performed before ICL V4 implantation to prevent intraocular hypertension and glaucoma. It is important to monitor changes in intraocular pressure (IOP) and the anterior chamber angle (ACA) after surgery.

Although gonioscopy and ultrasonic biomicroscopy can provide direct observation of the ACA, they share the drawback of being contact procedures. As a widely applied non-contact procedure used in the clinic, the Pentacam system (Oculus Inc., Wetzlar, Germany) allows measurement of the ACA, anterior chamber volume (ACV), axial anterior chamber depth (ACD) and ICL vault. ACV and ACD measurements obtained from the Pentacam are more useful in screening for angle closure, because they are less dependent on the configuration of the peripheral part of the ACA [1]. Moreover, the ACV has been described as a sensitive parameter for monitoring ACA width and LPI efficacy [2, 3].

The exact changes in the ACV after ICL implantation with an LPI cannot be determined from previous studies. A majority of studies involving ICL implantation have focused on changes in postoperative central vault. Since postoperative ACA was determined not only by central vault but also by iris root thickness and iris curvature, ACV is a useful parameter for a more comprehensive assessment of anterior chamber after ICL implantation. In the current study, we investigated the ACV, ACA, ACD and central vault after ICL implantation for the management of high myopia. In addition, we characterized ACV changes and the correlating factors after ICL implantation.

## Methods

### Patients

In this study, 45 eyes of 26 Chinese patients (8 males and 18 females) with a mean age of 32.47 ± 8.67 years (range 20–47 years) were assessed. The average preoperative spherical equivalent (SE) of all patients was − 15.27 ± 4.61 diopters (D). All patients were relatively healthy with no systemic diseases such as kidney diseases, hematologic diseases, immune diseases or a history of drug use. The Institutional Review Board at the Shanghai Ninth People’s Hospital, Shanghai JiaoTong University School of Medicine approved the study, and the study was performed in accordance with the Declaration of Helsinki. All patients signed an informed consent form.

Indications for ICL implantation included myopia of at least − 5.0 D, stable refraction for at least 1 year before surgery, 20 years of age or older, no pre-existing ocular pathologic features, no previous ocular surgery, IOP between 10 and 21 mmHg, corneal endothelial cell density (ECD) of more than 2000 cells/mm^2^, an ACA greater than grade III by gonioscopy and a clear crystalline lens.

### Preoperative examination

Before refractive surgery, all patients underwent a complete ophthalmic examination, including the logarithm of the minimal angle of resolution (logMAR) of the uncorrected visual acuity (UCVA), the logMAR of the best spectacle-corrected visual acuity (BSCVA), SE, ACV (Pentacam; Oculus, Wetzlar, Germany), ECD (Topcon-SP; Tokyo, Japan), corneal topography, slit-lamp microscope evaluation, biometry (IOL Master; Carl Zeiss Meditec, Jena, Germany) measurements and a dilated fundus evaluation. White-to-white diameter (WTW) using a surgical compass was also measured in ICL patients. Corneal horizontal diameter and axial length were measured by the IOL-Master.

### Surgical implantation of the collamer lens

To avoid postoperative pupillary block, which may result from ICL insertion, a single peripheral laser iridotomy at 12:00 o’clock (1 × 1 mm^2^) was performed 2 to 3 days before surgery. In all patients, 0.5% levofloxacin was topically applied for 3 days preoperatively. One hour before surgery, all pupils were dilated with cycloplegic agents (tropicamide and phenylephrine, Mydrin P; Santen, Osaka, Japan). Topical anesthesia was applied three times, 30 min before surgery, using 4% oxybuprocaine eye drops. ICLs were placed in the lens insertion cartridge under direct visualization using an operating microscope. A lid speculum was placed, a 3.2 mm temporal and vertical clear corneal incision was performed, and sodium hyaluronate (CP, Shandong, China) was injected into the anterior chamber. The injector tip was then placed on the incision, and the lens was slowly injected anterior to the iris plane to ensure proper orientation. Pupillary constriction was induced by acetylcholine injection into the anterior chamber. The remaining viscoelastic material was removed with gentle irrigation and washing with the injector. After surgery, topical tobramycin-dexamethasone eye ointment was applied.

### Postoperative follow-up

**Fig. 1:**
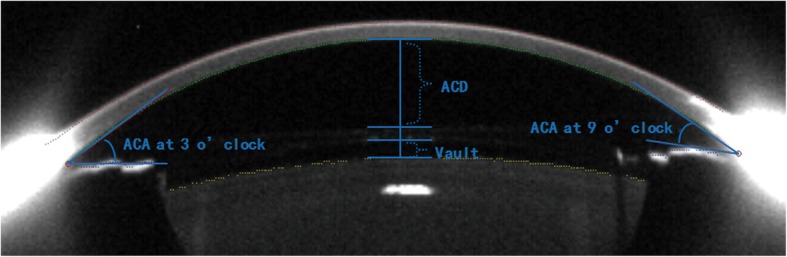
Postoperative image of the anterior segment taken by the Pentacam at the horizontal meridian. The anterior chamber angle (ACA) of the temporal and nasal quadrants (at 9:00 o’clock and 3:00 o’clock) and the anterior chamber volume (ACV) were automatically measured by the device’s software. The postoperative anterior chamber depth (ACD) was defined as the distance between the central posterior corneal endothelium and the anterior implantable contact lens (ICL) surface. The vault was measured as the central distance between the posterior ICL surface and the anterior crystalline lens capsule

The mean follow-up time was 4.28 months (3–6 months). The assessed outcome parameters included the logMAR of the UCVA and BSCVA at the last visit, refraction, ECD, lens vault and silt-lamp examination for lens transparency and inflammation. In addition, a Pentacam was used to observe ACD, ACV and the position of the ICL. As shown in Fig. 1, the ACV and ACA were automatically measured and calculated by the Pentacam. ACV was defined by Pentacam software as the volume of the anterior chamber from endothelium down to iris and lens evaluated in a zone of 12 mm around the anterior corneal apex. The ACD and vault were manually identified from images scanned by the Pentacam. The same clinician operated Pentacam and recorded the average value of 3-time measurements for each examination. Postoperative outcome data at 3 months after surgery was collected and analyzed.

### Statistical analysis

The data were analyzed using SPSS, version 22.0 for Windows (SPSS, Chicago, IL, USA). The normality of the samples was determined using the Kolmogorov–Smirnov test and homoscedasticity was determined using Levene’s test. The Kruskal–Wallis test was used for the comparison of several independent samples. The paired samples Wilcoxon signed-rank test was used for the comparison of two related samples. Pearson’s correlation test was used when samples fit the normal distribution; otherwise Spearman’s correlation test was performed. Generalized estimating equation (GEE) model adjusting within-patient intereye correlations was used to determine the correlative parameters of postoperative ACV. A two-tailed value of *P* < 0.05 was considered to be statistically significant.

## Results

Successful implantation was achieved in all patients. No complications occurred during the surgical procedures or follow-up period. None of the surgical cases required a second surgical procedure or prolonged topical medication. Visual acuity, SE, ACV, ACA, ACD and ICL central vault were demonstrated in Table 1.

**Table 1:** Demographics of the study population undergoing implantable contact lens (ICL) implantation (x ± SD)

Variant	Preoperative	Postoperative	*Z*	*P*
logMAR UCVA	1.56 ± 0.45	0.20 ± 0.17	5.848	< 0.001
Spherical equivalent (D)	−15.27 ± 4.61	−0.66 ± 1.14	5.842	< 0.001
ACV (mm^3^)	198.33 ± 33.08	118.65 ± 17.70	5.841	< 0.001
	range, 145–270	range, 81–150		
ACA at 3:00 o’ clock (°)	43.23 ± 5.72	27.44 ± 5.24	5.841	< 0.001
	range, 32.1–55.4	range, 15.5–41.3		
ACA at 9:00 o’ clock (°)	42.40 ± 5.81	26.82 ± 4.73	5.841	< 0.001
	range, 34.7–56.6	range, 16.1–39.0		
ACD (mm)	3.24 ± 0.25	2.32 ± 0.27	5.842	< 0.001
	range, 2.82–3.81	range, 1.54–2.78		
Vault (μm)		460 ± 250		
		range, 130–1110		

Data are expressed as means ± standard deviation

*UCVA* Uncorrected visual acuity, *D* Diopters, *ACV* Anterior chamber volume, *ACA* Anterior chamber angle, *ACD* Anterior chamber depth, *Z* Z value for Wilcoxon signed-rank test between preoperative and postoperative parameters, *P P* value for Wilcoxon signed-rank test between preoperative and postoperative parameters

After surgery, ACV significantly decreased by 79.68 mm^3^ (40.18%); ACD decreased by 0.92 mm (28.40%); ACA at 3 and 9 o’clock respectively decreased by 15.80°(34.70%) and 15.58°(36.75%).

**Fig. 2:**
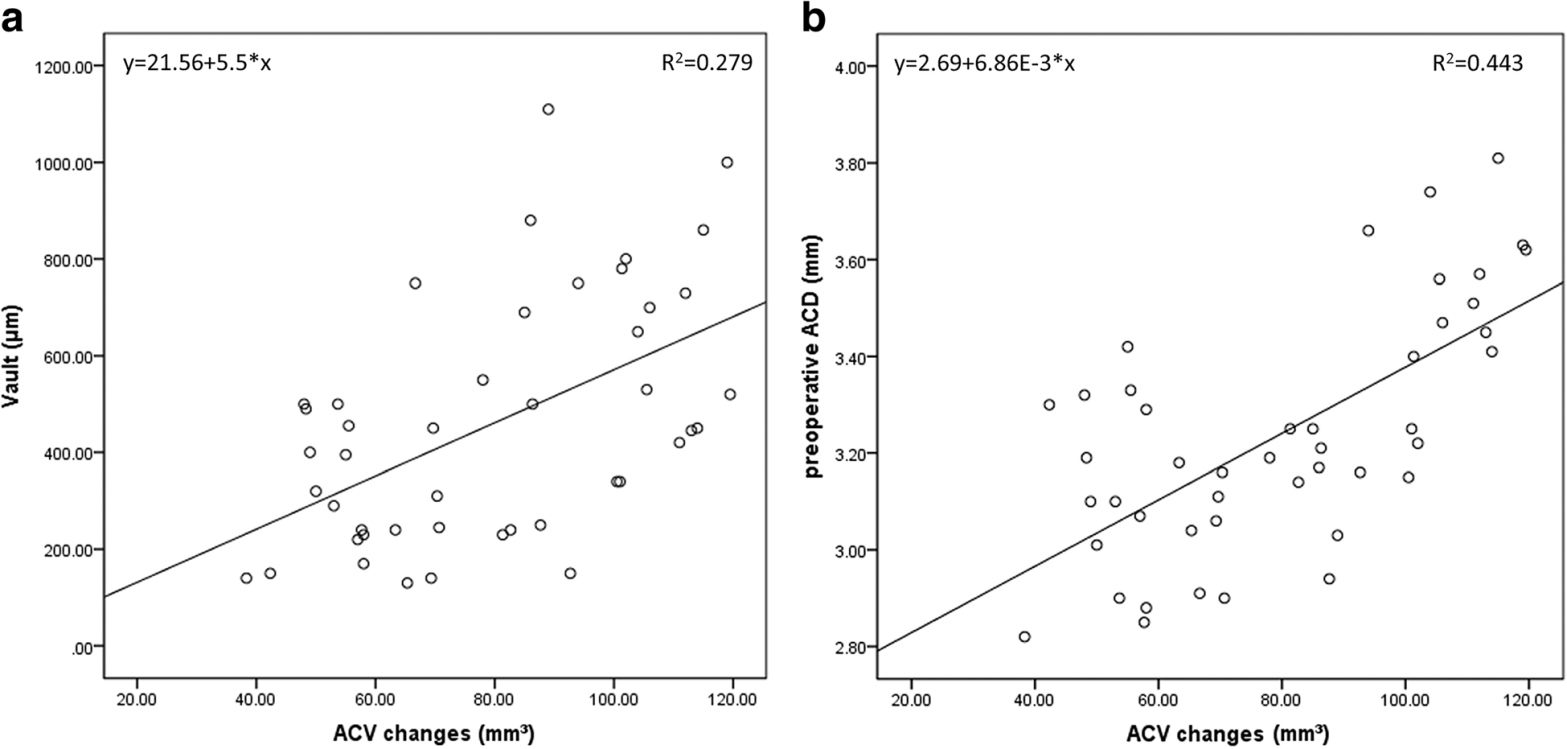
**a** A scatterplot revealed a statistically significant correlation between ACV change value (mm^3^) and ICL central vault (μm) (Pearson’s correlation coefficient *r* = 0.528, *P* < 0.001). Linear regression equation: y = 21.56 + 5.5*x. **b** Spearman’s correlation analysis revealed a positive and significant correlation between ACV change value (mm^3^) and preoperative ACD (mm) (*r* = 0.665, *P* < 0.001). Linear regression equation: y = 2.69 + 0.00686*x

**Fig. 3:**
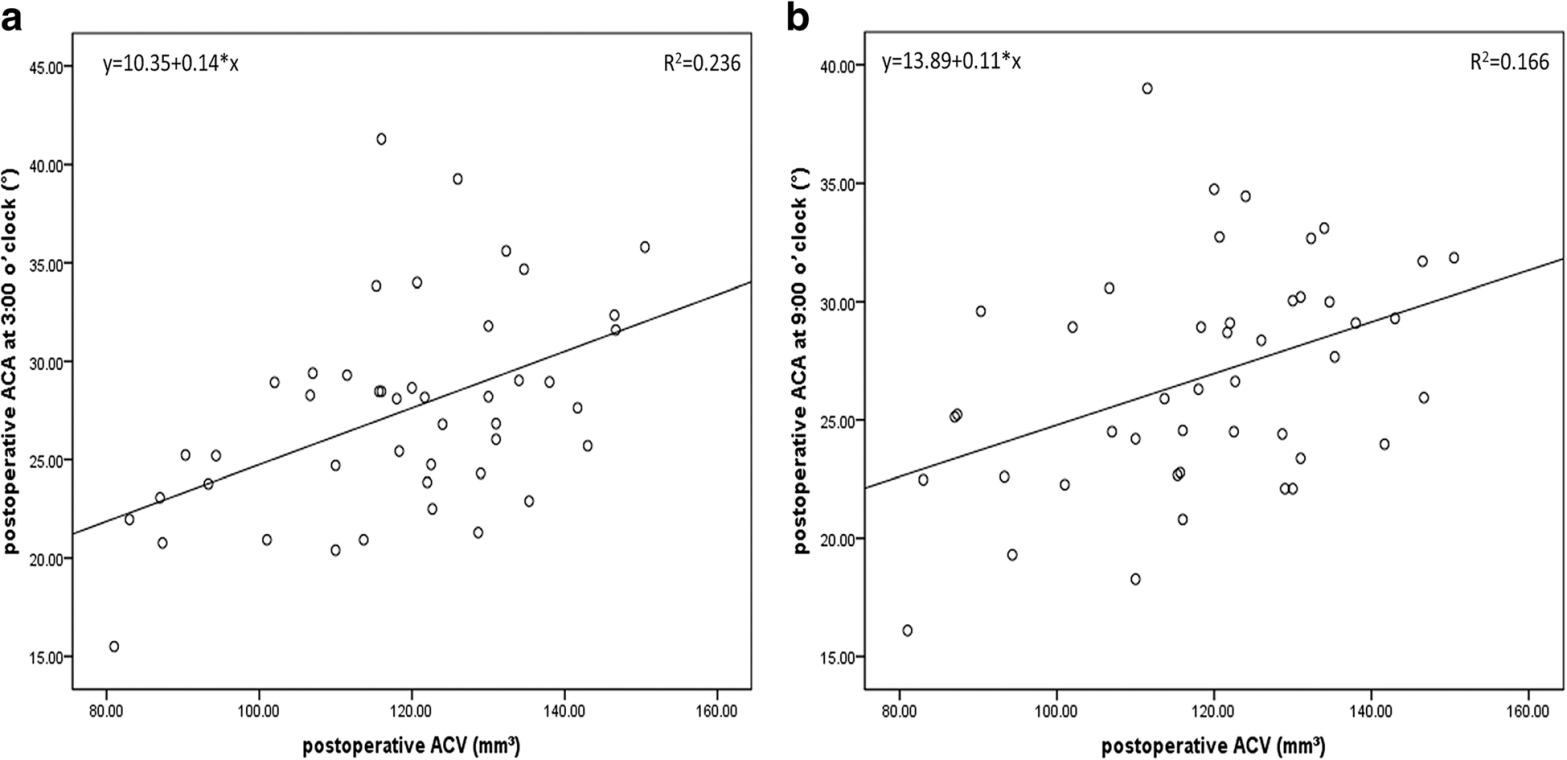
Scatterplots revealed statistically significant correlations between postoperative ACV and postoperative ACA at 3:00 o’clock (Spearman’s correlation *r* = 0.448, *P* = 0.002) (**a**) and at 9:00 o’clock (Spearman’s correlation *r* = 0.405, *P* = 0.006) (**b**). Linear regression equations were: y = 10.35 + 0.14*x (**a**) and y = 13.89 + 0.11*x (**b**)

Scatterplots showed positive correlations between ACV change value and central vault (*r* = 0.528, *P* < 0.001) and between ACV change value and preoperative ACD (*r* = 0.665, *P* < 0.001) (Fig. 2). Positive correlations between postoperative ACV and postoperative ACA at 3:00 o’clock (*r* = 0.448, *P* = 0.002) and 9:00 o’clock (*r* = 0.405, *P* = 0.006) were showed in Fig. 3.

Generalized estimating equation (GEE) model of postoperative ACV adjusting for within-patient intereye correlations was performed using preoperative ACV, preoperative ACD, preoperative ACA and central vault as independent variables.

The following GEE model was obtained:

$$ \mathrm{y}=0.21{8\upchi}_1+0.03{0\upchi}_2+0.66{9\upchi}_3-0.03{8\upchi}_4-30.217 $$

where y, × 1, × 2, × 3 and × 4 are the postoperative ACV (mm^3^), preoperative ACV (mm^3^), preoperative ACD (μm), preoperative ACA(°) (automatically measured by Pentacam defined as the smaller value of horizontal anterior chamber angles) and ICL central vault (μm) respectively. Detailed information were demonstrated in Table 2. The predicted postoperative ACV values calculated from the deduced model were in agreement with the measured values (Pearson’s correlation coefficient *r* = 0.886, *P* < 0.001).

**Table 2:** Factors predicting postoperative ACV determined by generalized estimating equation model adjusting for within-patient intereye correlations

Independent variable	Coefficient	SE	OR	*P*
Preoperative ACV (mm^3^)	0.218	0.0694	1.244	0.002
Preoperative ACD (μm)	0.030	0.0096	1.030	0.002
Preoperative ACA (°)	0.669	0.2387	1.952	0.005
Vault (μm)	−0.038	0.0076	0.962	< 0.001

*SE* Standard error, *OR* Odds ratio, *P* Significance probability, *ACV* Anterior chamber volume, *ACD* Anterior chamber depth, *ACA* The smaller value of horizontal anterior chamber angles automatically determined by Pentacam

## Discussion

The results of current study showed a decrease of ACV after ICL implantation. The change value of ACV was positively correlated with vault and preoperative ACD. It can be seen from the regression model that postoperative ACV significantly positively correlated with preoperative ACV, ACD and horizontal ACA and negatively correlated with ICL central vault. Complementary to vault and ACD, ACV is a sensitive parameter for postoperative monitoring and complications predicting after ICL implantation.

The change in postoperative ACV was the result of two contrary effects on anterior segment structure changes: one was an increase in ACV caused by iris flattening after LPI, and the other was a decrease in ACV caused by forward movement of the iris under the push from the ICL. Unterlauft and colleagues showed that the mean ACV increased from 48.2 ± 3.6 to 60.6 ± 2.4 mm^3^ in acute angle closure eyes and from 60.4 ± 4.6 to 74.1 ± 3.7 mm^3^ in fellow eyes after LPI [4]. LPI can lead to iris flattening, angle widening and an increase in ACV in eyes with and without angle closure [4–7]. However, our study showed a 40.2% decrease in ACV, even after LPI, indicating that the decrease in the ACD after ICL insertion plays a more dominant role in the change in ACV than does LPI. A study conducted on healthy Chinese adults reported that the ACV was correlated with most of the anterior segment parameters, especially ACD, which accounted for approximately 85% of the variation in the ACV [8], consistent with the findings in the current study.

Previous studies suggested that the ACV is the strongest determinant and the most sensitive parameter among all of the established and newly identified factors associated with angle width [1–3, 9, 10]. A smaller ACV was reported to be independently associated with narrow angles, even after controlling for other known ocular risk factors, and it performs better than the ACD as a screening parameter for narrow angles [10]. Another Chinese Singaporean population-based study showed that the strongest determinants for angle width among anterior segment optical coherence tomography (ASOCT) and A-scan independent variables were the ACV, followed by anterior chamber area (the cross-sectional area of the ACV) [2]. Compared with Caucasians, ethnic Chinese individuals have a smaller ACV (independent of ACD, anterior chamber width, iris curvature, iris area, pupil diameter, corneal radius of curvature and axial length) [9], which may explain the higher rate of angle closure in the Chinese population. The ACV was the most prominent contributor to angle width variation in both Chinese and Caucasians in that previous study [9]. Lam et al. reported that middle-aged female Chinese subjects are more likely to have angle closure than are their male counterparts. Female subjects had a smaller ACV but a similar ACD compared with those observed in male subjects. Thus, they concluded that the ACV is a more sensitive parameter to screen for a crowded anterior chamber than is a linear measurement of the ACD [3]. One explanation for this phenomenon may be that eyes with similar ACDs differ in their corneal radius of curvature, peripheral iris thickness and/or iris curvature, which contributes to angle width. Information provided by ACD evaluation is quite limited, because the ACD is a measure along just one axis. The eye is not perfectly spherical; therefore, the measured parameters may be influenced by the measurement plane, whereas the ACV is a more comprehensive parameter of anterior chamber evaluation involving corneal and iris morphologies and allowing for a three-dimensional assessment.

Moreover, the ACV is the only parameter that changed significantly after LPI, and it has the potential to be used as a numerical proxy for iris position when evaluating and monitoring patients after LPI [11–13]. Previous studies have revealed that LPI can lead to iris flattening, angle widening and an increase in the ACV in eyes with and without angle closure [4–7]. However, LPI was not effective in all eyes throughout the follow-up period [14]. He et al. reported that approximately 60% of Chinese eyes with primary angle closure suspects (PACS) exhibited persistent appositional closure in at least one quadrant after LPI [15], and Ang et al. reported that approximately 11% of Caucasian eyes with PACS were closed in at least one quadrant after LPI [16]. Ethnic and individual differences in iris thickness and flexibility likely account for the varying responses to LPI [17]. Therefore, ACV is a useful and necessary parameter that can help identify eyes that respond poorly to LPI and monitor the efficacy of LPI throughout the postoperative follow-up period after ICL implantation.

Despite a 40.18% decrease in ACV after ICL implantation, no angle closure or narrow angle condition required intervention in our study. Several previous studies have reported threshold values for discriminating between healthy eyes and eyes with narrow angles using the Pentacam system. The threshold values reported from a German study were 90.5 mm^3^ for ACV, 2.1 mm for ACD and 27.25° for ACA [18]. In another study from Italy, the threshold values were 84 mm^3^ for ACV, 1.93 mm for ACD and 22.4° for ACA [19]. The threshold values from an Iran study were 100 mm^3^ for ACV, 2.1 mm for ACD and 26° for ACA [20]. In our study, the postoperative values were 118.65 mm^3^ for ACV and 2.32 mm for ACD, which are higher than those previously reported threshold values. For eyes with postoperative parameter values under the threshold detected by Pentacam, additional examinations, such as gonioscopy and ultrasonic biomicroscopy, should be appointed.

Our results showed a positive correlation between postoperative ACV and horizontal ACA, it provided clinical evidence of postoperative monitoring value of ACV after ICL implantation. As preoperative ACD positively correlates with postoperative ACV, ICL lens of larger diameter could be considered in condition of large preoperative ACD. A negative correlation was detected between postoperative ACV and central vault as expected. Since postoperative ACV reflected iris root thickness, iris curvature and peripheral vault in addition to central vault, a more comprehensive anterior chamber assessment can be achieved by including ACV as a complementary parameter besides central vault and ACD.

There are some limitations to this study. Firstly, ACV, ACD and AVA can also be determined by pupil diameter and accommodation status. Measurements were taken in the non-mydriatic state, and the room lighting conditions were kept constant. Moreover, the internal fixation target of Pentacam may not provide good accommodation control, especially for young subjects with relatively more active accommodation. In addition, based on an accurate measurement of the IOP, the relationship between ACV changes and IOP after ICL surgery requires further investigation. Thus, in future studies, a large-scale sample study with a long-term follow-up period is needed.

## Conclusions

In conclusion, this study found that, in addition to vault and ACD, ACV is a sensitive parameter with certain value of preoperative assessment and postoperative monitoring in ICL implantation. Further studies are needed to confirm its predictive application in the diagnosis and treatment of glaucoma.

## Abbreviations

ACA: Anterior chamber angle
ACD: Anterior chamber depth
ACV: Anterior chamber volume
ASOCT: Anterior segment optical coherence tomography
BSCVA: Best spectacle-corrected visual acuity
D: Diopters
ECD: Endothelial cell density
ICL: Implantable contact lens
IOP: Intraocular pressure
LPI: Laser peripheral iridectomy
PACS: Primary angle closure suspects
SE: Spherical equivalent
UCVA: Uncorrected visual acuity
VIF: Variance inflation factor
WTW: White-to-white diameter
